# Supplementary material for: Timing Is Everything: The Metabolic Partitioning of Suberin-Destined Carbon
Source: Plants (Basel). 2025 May 10;14(10):1433. doi: 10.3390/plants14101433 (PMC12114950; doi:10.3390/plants14101433)
Supplement: Supplementary file 1 [file plants-14-01433-s001.zip › plants-3590428-supplementary.pdf]

Article

Timing Is Everything: The Metabolic Partitioning of Suberin-Destined Carbon

Jessica L. Sinka and Mark A. Bernards *

Department of Biology, Western University, London, ON N6A 5B7, Canada; jsinka2@uwo.ca
* Correspondence: bernards@uwo.ca

Supplementary Materials:

Table S1: Tukey's Pairwise Comparison was performed for data shown in figure 3A where the depletion of 13C label in glucose overtime, following a single bolus application of [$^{13}C_6$]-glucose, was assessed. P-values for comparisons of the proportion of glucose with ^{13}C label at 0-, 24-, 48-, and 72-hpw are presented (n = 5).

**hour post wounding**			
	24	48	72
0	<0.0001	<0.0001	<0.001
24		0.0175	0.0273
48			0.996

Table S2: Tukey's Pairwise Comparison was performed for data shown in figure 3B where the sucrose pool was enriched with 13C label overtime, following a single bolus application of [$^{13}C_6$]-glucose. P-values for comparisons of the proportion of sucrose with ^{13}C label at 0-, 24-, 48-, and 72-hpw are presented (n = 5).

**hour post wounding**			
	24	48	72
0	0.0126	0.9273	0.5139
24		0.0425	0.0007
48			0.2231

Table S3: Tukey's Pairwise Comparison was performed for data shown in figure 5A where the proportional ^{13}C enrichment of proxy metabolites, grouped by timepoint, were compared. Proxy metabolites were assessed at 0-, 24-, 48-, and 72- hpw following a single bolus application of [$^{13}C_6$]-glucose at the time of wounding (i.e., 0 hpw). P-values for comparisons of the proportion ^{13}C in proxy metabolites are presented (n = 5).

**0 hours post wounding**				
	Shikimate	L-Phenylalanine	Palmitate	Stearate
Citrate	0.1959	>0.9999	>0.9999	>0.9999
Shikimate		0.1859	0.1718	0.177
L-Phenylalanine			>0.9999	>0.9999
Palmitate				>0.9999
**24 hours post wounding**				
	Shikimate	L-Phenylalanine	Palmitate	Stearate
Citrate	<0.0001	<0.0001	0.9263	0.9069
Shikimate		0.2961	<0.0001	<0.0001
L-Phenylalanine			<0.0001	<0.0001
Palmitate				>0.9999
**48 hours post wounding**				
	Shikimate	L-Phenylalanine	Palmitate	Stearate
Citrate	<0.0001	<0.0001	0.4608	0.4233
Shikimate		0.9513	<0.0001	<0.0001
L-Phenylalanine			<0.0001	<0.0001
Palmitate				>0.9999
**72 hours post wounding**				
	Shikimate	L-Phenylalanine	Palmitate	Stearate
Citrate	<0.0001	<0.0001	0.2494	0.2308
Shikimate		0.9978	<0.0001	<0.0001
L-Phenylalanine			<0.0001	<0.0001
Palmitate				>0.9999

Table S4: Tukey's Pairwise Comparison was performed for data shown in figure 5B where the amount of ^{13}C enriched compound (ug/ mg tissue), grouped by timepoint, were compared. Proxy metabolites were assessed at 0-, 24-, 48-, and 72- hpw, following a single bolus application of [$^{13}C_6$]-glucose at the time of wounding (i.e., 0 hpw). P-values for comparisons of the amount of ^{13}C in proxy metabolites are presented (n = 5).

**0 hours post wounding**				
	Shikimate	L-Phenylalanine	Palmitate	Stearate
Citrate	0.4106	0.9869	0.983	0.9853
Shikimate		0.1935	0.1818	0.1883
L-Phenylalanine			>0.9999	>0.9999
Palmitate				>0.9999
**24 hours post wounding**				
	Shikimate	L-Phenylalanine	Palmitate	Stearate
Citrate	0.0001	0.1305	0.0289	0.0264
Shikimate		<0.0001	<0.0001	<0.0001
L-Phenylalanine			0.9401	0.928
Palmitate				>0.9999
**48 hours post wounding**				
	Shikimate	L-Phenylalanine	Palmitate	Stearate
Citrate	<0.0001	0.0042	0.0007	0.0006
Shikimate		<0.0001	<0.0001	<0.0001
L-Phenylalanine			0.9211	0.9093
Palmitate				>0.9999
**72 hours post wounding**				
	Shikimate	L-Phenylalanine	Palmitate	Stearate
Citrate	<0.0001	0.0009	0.0004	0.0003
Shikimate		<0.0001	<0.0001	<0.0001
L-Phenylalanine			0.9937	0.991
Palmitate				>0.9999

Table S5: One-sample T-Tests were conducted on all compounds suspected for ^{13}C enrichment, as seen in figure 3 & 5. Tissue was sampled at 0-, 24-, 48-, and 72- hpw following a single application of $^{13}C_6$-glucose. Proportion of ^{13}C label incorporation into proxy metabolites was tested against a theoretical mean of 0.

Hour post wounding	Compound name	Mean	SD	df	T	P
0	Glucose	0.4108	0	4		
	Sucrose	0.1243*	0.07922	4	3.509	0.0247
	Citrate	0.001595	0.001758	4	2.028	0.1124
	Shikimate	0.04859	0.07319	4	1.485	0.2118
	L-Phenylala-nine	0.00096	0.00107	4	2.006	0.1153
	Palmitate	1.71E-05	3.82E-05	4	1.000	0.3739
	Stearate	0.0003704	0.0008282	4	1.000	0.3739
24	Glucose	0.06633****	0.009315	4	15.92	<0.0001
	Sucrose	0.2989**	0.1147	4	5.826	0.0043
	Citrate	0.01573*	0.008793	3	3.578	0.0373
	Shikimate	0.1724**	0.05729	4	6.731	0.0025
	L-Phenylala-nine	0.1358***	0.02889	4	10.51	0.0005
	Palmitate	0.001079**	0.0004778	4	10.51	0.0072
	Stearate	0	0	4		
48	Glucose	0.1036***	0.02236	4	5.285	0.0005
	Sucrose	0.1543**	0.06529	4	5.285	0.0062
	Citrate	0.01329**	0.005842	4	5.087	0.007
	Shikimate	0.09389***	0.02011	4	10.44	0.0005
	L-Phenylala-nine	0.09927***	0.01659	4	13.38	0.0002
	Palmitate	0.0005053	0.0006675	4	1.693	0.1658
	Stearate	0	0	4		
72	Glucose	0.1012***	0.02492	4	9.079	0.0008
	Sucrose	0.0553**	0.02017	4	6.131	0.0036
	Citrate	0.02324***	0.00532	4	9.769	0.0006
	Shikimate	0.0924**	0.02698	4	7.658	0.0016
	L-Phenylala-nine	0.09571**	0.02698	4	8.230	0.0012
	Palmitate	0.0005156	0.0007133	4	1.616	0.1813
	Stearate	0	0	4		

Table S6: Simple linear regression of depletion of ^{13}C label in glucose over 6 hours. The slopes of the lines are significantly different (F = 5.425, DFd = 216, P=0.0013) from one another (n=10-15).

Time of label application (h):	Y = mx+b	95% CIs Slope	R^2 (Goodness of fit)
0	Y = -0.02420X + 0.1845	-0.03500 to -0.01340	0.2576
24	Y = -0.05482X + 0.3715	-0.06794 to -0.04170	0.5467
48	Y = -0.05399X + 0.4077	-0.06538 to -0.04260	0.6125
72	Y = -0.03960X + 0.3894	-0.05638 to -0.02282	0.345

Table S7: One-sample T-Tests were conducted on the proportion of 13C label remaining in glucose after allowing the tissue to metabolize for 6 hours post exogenous label application, as seen in figure 6. Multiple bolus applications of $^{13}C_6$-glucose were applied throughout to wound-healing tissue (i.e., 0-, 24-, 48-, and 72- hpw). Proportion of ^{13}C label incorporation into proxy metabolites was tested against a theoretical mean of 0.

Time of label application (h)	Time post label application (h)	Mean	SD	df	T	P
0	0	0.2175	0	14		
	1	0.1747****	0.04806	14	14.08	<0.0001
	3	0.1381****	0.06222	14	8.594	<0.0001
	6	0.06541****	0.0381	14	6.649	<0.0001
24	0	0.383	0	14		
	1	0.3066****	0.09828	14	12.08	<0.0001
	3	0.2009****	0.1083	14	7.187	<0.0001
	6	0.04732**	0.04673	14	3.922	0.0015
48	0	0.4405	0	14		
	1	0.3366****	0.1381	14	9.440	<0.0001
	3	0.2088****	0.1105	14	7.066	<0.0001
	6	0.1074****	0.07295	14	5.702	<0.0001
72	0	0.4025	0	14		
	1	0.3358***	0.2031	9	5.230	0.0005
	3	0.2738***	0.1444	9	5.998	0.0002
	6	0.1622***	0.09485	9	5.408	0.0004

Table S8: Tukey's Pairwise Comparison was performed for data shown in figure 7 where the proportional ^{13}C enrichment of sucrose, grouped by timepoint of label application, were compared. Multiple bolus applications of [$^{13}C_6$]-glucose were applied to wound-healing tissue at 0-, 24-, 48-, and 72- hpw. P-values are presented for comparisons of (left) the proportion of sucrose with ^{13}C label and (right) the amount of 13C enriched compound from tissue sampled 3 hours post label application (n = 10-15).

Proportion:

**hour post wounding (h)**	**24**	**48**	**72**
**0**	<0.0001	<0.0001	0.0351
**24**		0.9761	0.2693
**48**			0.4534

Amount:

**hour post wounding (h)**	**24**	**48**	**72**
**0**	0.4806	0.2956	0.0678
**24**		0.0860	0.0144
**48**			0.04806

Table S9: Tukey's Pairwise Comparison was performed for data shown in figure 8A where the proportional ^{13}C enrichment of proxy metabolites, grouped by timepoint of label application, were compared. Multiple bolus applications of [$^{13}C_6$]-glucose were applied to wound-healing tissue (i.e., 0-, 24-, 48-, and 72- hpw). P-values are presented for comparisons of the proportion of proxy metabolites with ^{13}C label from tissue sampled 3 hours post label application (n = 9-15).

**0 hours post wounding**				
	Shikimate	L-Phenylalanine	Palmitate	Stearate
Citrate	0.991	0.0832	0.0157	0.0254
Shikimate		0.0278	0.0037	0.0064
L-Phenylalanine			0.9954	0.9996
Palmitate				0.9997
**24 hours post wounding**				
	Shikimate	L-Phenylalanine	Palmitate	Stearate
Citrate	<0.0001	>0.9999	0.0012	0.0065
Shikimate		<0.0001	<0.0001	<0.0001
L-Phenylalanine			0.0028	0.0128
Palmitate				0.9792
**48 hours post wounding**				
	Shikimate	L-Phenylalanine	Palmitate	Stearate
Citrate	0.9496	0.0033	0.1085	0.513
Shikimate		0.0124	0.0078	0.1013
L-Phenylalanine			<0.0001	<0.0001
Palmitate				0.8655
**72 hours post wounding**				
	Shikimate	L-Phenylalanine	Palmitate	Stearate
Citrate	0.9588	0.1875	0.0008	0.0013
Shikimate		0.0055	0.0002	0.0003
L-Phenylalanine			<0.0001	<0.0001
Palmitate				0.9997

Table S10: Tukey's Pairwise Comparison was performed for data shown in figure 8B where the proportional ^{13}C enrichment of proxy metabolites, grouped by timepoint of label application, were compared. Multiple bolus applications of [$^{13}C_6$]-glucose were applied to wound-healing tissue (i.e., 0-, 24-, 48-, and 72- hpw). P-values are presented for comparisons of the amount of 13C enriched compound from tissue sampled 3 hours post label application (n = 9-15).

**0 hours post wounding**				
	Shikimate	L-Phenylalanine	Palmitate	Stearate
Citrate	0.0089	<0.0001	<0.0001	<0.0001
Shikimate		0.5327	0.4307	0.4714
L-Phenylalanine			>0.9999	>0.9999
Palmitate				>0.9999
**24 hours post wounding**				
	Shikimate	L-Phenylalanine	Palmitate	Stearate
Citrate	0.0001	<0.0001	<0.0001	<0.0001
Shikimate		<0.0001	<0.0001	<0.0001
L-Phenylalanine			0.9993	>0.9999
Palmitate				0.9997
**48 hours post wounding**				
	Shikimate	L-Phenylalanine	Palmitate	Stearate
Citrate	0.1488	0.0407	0.0045	0.0145
Shikimate		<0.0001	<0.0001	<0.0001
L-Phenylalanine			0.9892	>0.9999
Palmitate				0.9925
**72 hours post wounding**				
	Shikimate	L-Phenylalanine	Palmitate	Stearate
Citrate	0.0001	0.7511	0.4771	0.5132
Shikimate		<0.0001	<0.0001	<0.0001
L-Phenylalanine			0.993	0.9964
Palmitate				>0.9999

Table S11: One-sample T-Tests were conducted on all compounds suspected for ^{13}C enrichment, as seen in figure 7 & 8. Multiple bolus applications of [$^{13}C_6$]-glucose were applied to wound-healing tissue (i.e., 0-, 24-, 48-, and 72- hpw). Proportion of ^{13}C label incorporation into proxy metabolites 3 hours post label application was tested against a theoretical mean of 0.

Hour post wounding	Compound name	Mean	SD	df	T	P
0	Sucrose	0.1288****	0.06776	13	7.113	<0.0001
	Citrate	0.1109**	0.1117	10	3.293	0.0081
	Shikimate	0.1269*	0.1619	10	2.600	0.0265
	L-Phenylalanine	0.01661*	0.04489	8	1.848	0.0335
	Palmitate	0.00373****	0.00198	10	6.247	<0.0001
	Stearate	0.00953***	0.005693	10	5.552	0.0002
24	Sucrose	0.09817****	0.09817	14	9.119	<0.0001
	Citrate	0.1019**	0.08288	10	4.078	0.0022
	Shikimate	0.2735****	0.08785	13	11.65	<0.0001
	L-Phenylalanine	0.09851**	0.07899	9	3.944	0.0034
	Palmitate	0.07899**	0.001704	14	3.236	0.006
	Stearate	0.0144**	0.01288	13	4.183	0.0011
48	Sucrose	0.1275****	0.06727	14	7.343	<0.0001
	Citrate	0.09436*	0.09482	9	3.147	0.0118
	Shikimate	0.1104****	0.07229	14	5.916	<0.0001
	L-Phenylalanine	0.2267**	0.1741	9	4.120	0.0026
	Palmitate	0.001162**	0.001232	12	3.402	0.0053
	Stearate	0.03197**	0.03197	13	4.161	0.0011
72	Sucrose	0.1694**	0.1269	9	4.224	0.0022
	Citrate	0.1696***	0.03114	5	12.18	0.0003
	Shikimate	0.1189****	0.07483	14	6.154	<0.0001
	L-Phenylalanine	0.2191***	0.1287	9	5.383	0.0004
	Palmitate	0.002582***	0.001704	9	4.792	0.001
	Stearate	0.007204	0.01254	9	1.817	0.1026

Table S12: Tukey's Pairwise Comparison was performed for data shown in figure 10 where individual suberin-derived phenolic and aliphatic monomers were assessed for changes in proportion of 13C label accumulation from independent applications of $^{13}C_6$-glucose during early wound-healing. $^{13}C_6$-glucose was applied to wound-healing tissue at 0-, 24-, 48-, and 72-hours post-wounding and allowed to wound-heal until 168 hpw. Replicates from each time of label application, were normalized to the average proportion of ^{13}C label found in glucose at the time of labelling (n = 9-15).

**Total Phenolics**			
Time of label application (h)	24	48	72
0	0.5673	0.0044	0.6717
24		0.1001	0.9970
48			0.0559
**18:1 Dioic acid**			
Time of label application (h)	24	48	72
0	0.6683	0.0105	<0.0001
24		0.1735	0.0013
48			0.2547
**18:1 ω-OH FA**			
Time of label application (h)	24	48	72
0	0.9985	0.0170	0.0001
24		0.0153	0.0001
48			0.3658
**C22:0 fatty acid**			
Time of label application (h)	24	48	72
0	0.0324	0.0063	0.0017
24		0.9358	0.7410
48			0.9731
**C22:0 alkanol**			
Time of label application (h)	24	48	72
0	0.9710	0.2384	0.1192
24		0.5018	0.2959
48			0.9725
**Ferulic acid**			
Time of label application (h)	24	48	72
0	0.9173	0.6895	0.0208
24		0.9730	0.1241
48			0.2645

Table S13: One-sample T-Tests were conducted on the proportion of 13C label in suberin monomers. Multiple bolus applications of $^{13}C_6$-glucose were applied to wound-healing tissue (i.e. 0-, 24-, 48-, and 72- hpw) which was left to wound-heal for 168h. Proportion of ^{13}C label incorporation into suberin monomers was tested against a theoretical mean of 0.

Time of label application (h)	Compound name	Mean	SD	df	T	P
0	Total phenolics	0.02489	0.01529	8	4.884	0.0012
	18:1 Dioic acid	0.04442	0.01685	14	10.21	<0.0001
	18:1 ω-OH FA	0.04687	0.02110	13	8.310	<0.0001
	C22:0 fatty acid	0.08005	0.04629	14	6.697	<0.0001
	C22:0 alkanol	0.08935	0.03910	14	8.851	<0.0001
	Ferulic acid	0.02755	0.01041	14	10.25	<0.0001
24	Total phenolics	0.03707	0.01871	9	6.265	0.0001
	18:1 Dioic acid	0.06536	0.04845	13	5.048	0.0002
	18:1 ω-OH FA	0.04301	0.03767	11	3.955	0.0023
	C22:0 fatty acid	0.1645	0.09802	12	6.084	<0.0001
	C22:0 alkanol	0.1007	0.05933	12	6.121	<0.0001
	Ferulic acid	0.03881	0.02455	12	5.699	<0.0001
48	Total phenolics	0.05819	0.02846	10	6.781	<0.0001
	18:1 Dioic acid	0.1043	0.03255	13	11.99	<0.0001
	18:1 ω-OH FA	0.1199	0.05417	12	7.982	<0.0001
	C22:0 fatty acid	0.1837	0.08863	13	7.473	<0.0001
	C22:0 alkanol	0.1376	0.07407	13	6.950	<0.0001
	Ferulic acid	0.04647	0.06512	13	2.670	0.0193
72	Total phenolics	0.03536	0.01489	10	7.854	<0.0001
	18:1 Dioic acid	0.1392	0.07935	13	6.566	<0.0001
	18:1 ω-OH FA	0.1594	0.1018	12	5.649	0.0001
	C22:0 fatty acid	0.1971	0.07976	12	8.909	<0.0001
	C22:0 alkanol	0.1492	0.09377	11	5.510	0.0002
	Ferulic acid	0.07811	0.05923	14	5.108	0.0002
